# Renal Metastases of a Femur Osteosarcoma: A Case Report and a Review of the Literature

**DOI:** 10.1155/2012/259193

**Published:** 2012-02-28

**Authors:** Yousra Akasbi, Samia Arifi, Karim Lahlaidi, Tarik Namad, Nawfel Mellas, Mohammed Jamal El Fassi, My Hassan Farih, Afaf Amarti, Omar El Mesbahi

**Affiliations:** ^1^Medical Oncology Department, Hassan II University Hospital, Fez, Morocco; ^2^Urology Department, Hassan II University Hospital, Fez, Morocco; ^3^Department of Pathology, Hassan II University Hospital, Fez, Morocco

## Abstract

This paper discusses a rare case of renal metastatic osteosarcoma. A 25-year-old man with a history of metastatic osteosarcoma involving his right kidney was referred to our institution for treatment. He was managed with chemotherapy. An exhaustive review of the English literature pertaining to this disease was performed. To our knowledge, this case represents only the sixteenth. The literature suggests that the incidence of renal involvement in osteosarcoma is significant and that the treatment should be multidisciplinary in such patients.

## 1. Introduction

Renal metastases from osteosarcoma are extremely rare. We present a case of a young male with osteosarcoma of the left femur who developed late recurrence in the form of large metastatic renal and pulmonary lesions. Review of the literature suggests that osteosarcoma metastases of the kidneys usually exhibit aggressive behaviour with poor prognosis. However, local and systemic relapses are possible, even 5 or more years since the beginning of treatment, a long-term followup is recommended. 

## 2. Case Report

A 25-year-old male was diagnosed in 2004 as a femur osteosarcoma with no evidence of distance metastases. Initial treatment was by resection of the primary tumour with adjuvant chemotherapy.

**Figure 1:**
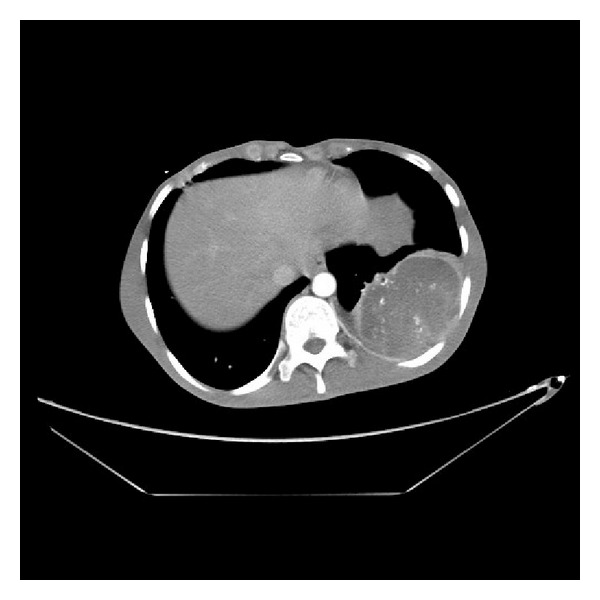
Abdominal computed tomography scan revealed renal metastases of osteosarcoma.

**Figure 2:**
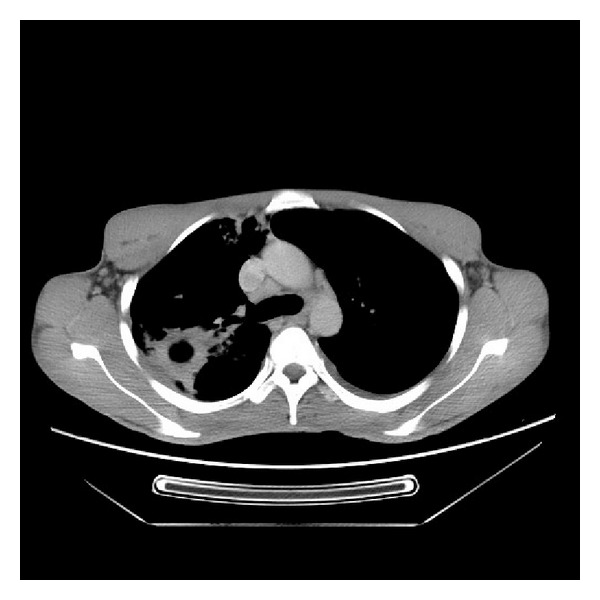
Chest computed scan revealed pleural metastases.

Thereafter, he remained well until April 2010 when he presented with a 6-week history of painless haematuria and painful masse in the lumbar region. Abdominal CT scan revealed a renal masse (Figure 1). This entity has not been previously described and the initial suspicion was that the patient had developed a further primary tumour. An ultrasound-guided fine needle biopsy confirmed the diagnosis of metastases. Chest computer scan showed pleural metastases (Figure 2).

 Preoperative chemotherapy based on API regimen (adriablastin 60 mg/m^2^ D1, ifosfamide 1,8 g/m^2^ D1–D5, mesna 1,8 g/m^2^ D1–D5, cisplatin 60 mg/m^2^ D2) is started. A thoracoabdominal computer tomography scan after the 3 cycles of chemotherapy demonstrated that the lesions enlarged rapidly, a second regimen of chemotherapy based on cisplatin and etoposide was programmed.

## 3. Discussion

Osteosarcoma is the most frequent malignant tumor of bones. The peak incidence occurs in the second decade of life, and the metaphyseal part of long bones is the site most frequently involved.

Classical high-grade osteosarcoma of the extremity has more of a tendency to metastasize, unlike low-grade parosteal osteosarcomas.

Primary osteosarcoma is a highly aggressive tumor that metastasizes by hematogenous dissemination. At diagnosis, nearly all patients will have microscopic metastases [1]. Despite resection and chemotherapy, 30%–40% of patients with localized disease will experience relapse, usually within 3 years [2]. In Our case, the patient experienced relapse 6 years after the treatment. Once this happens, the overall survival ranges from 13% to 57% [3, 4].

The lung is the most common site of metastatic disease, and the rarer instances of soft tissue and solid organ metastases have been considered preterminal, however, extrapulmonary sites are increasingly affected in treated patients. This may be because of change in the natural history of the disease by multiagent chemotherapy or longer survival times of these patients [1, 5]. In fact, the most commonly affected extrapulmonary site is the skeletal system followed by brain, liver, pelvis, and soft tissues. In the current case, kidney is affected.

The reported incidence of renal metastasis of extrarenal neoplasms varies from 2 to 20% [6]. Renal metastasis of osteosarcoma is usually detected after death as part of widespread disease 10–12% of patient autopsies showed renal involvement [7, 8]. Whereas 15% of patients will have clinically detectable lung metastases at diagnosis, renal metastases are usually silent [1].

Premortem diagnosis of renal metastasis is rare [1]. An intensive review of the literature demonstrates only 16 cases of premortem diagnosis of renal involvement among patients with osteosarcoma [5, 9–11].

When osteosarcoma of the kidney is discovered, metastatic disease from a bone primary is more likely than a primary lesion. Also, the presentation of these entities tends to differ.

Previous studies suggested that renal metastases of osteosarcoma were usually detected 2.2 years after the primary diagnosis. However, a more recent literature review found a mean interval of 62 months from time of treatment of primary tumor to diagnosis of renal metastases [12].

Hallet et al. reported on a patient whose renal metastasis was found 14 years after treatment of the primary tumor [13]. However, as demonstrated by our case and that of Ayres et al., these lesions have the potential to enlarge rapidly [14].

The earliest report of renal involvement by osteosarcoma is likely from a case involving a 15-year-old girl described by Weber in 1931 [15].

Most surveillance protocols after primary resection of osteosarcoma involve radiographic imaging of the bones and lungs. The abdomen is not routinely evaluated. Before the development of ultrasonography and CT, renal lesions in osteosarcoma were detected by intravenous pyelogram or, rarely, as areas of increased calcification on abdominal radiographs [16]. Unfortunately, in contrast with primary renal osteosarcoma, metastatic lesions usually do not possess enough calcification to be seen on simple radiographs [17]. In the contemporary setting, it is likely that most renal metastases will be detected by CT. Interestingly, however, a previous report found a patient's disease was missed by radiograph and CT and detected only by an abnormality on bone scan [13].

The diagnosis of metastatic disease should be made by imaging because some authors have expressed concern that needle biopsies risk dissemination of disease that is likely to be chemoradiation therapy resistant [18].

FDG-PET is sometimes used to confirm suspected pulmonary metastases seen on CT. Some believe that this modality is useful in detecting distant recurrences, especially in patients with extensive surgical histories or previous radiation therapy [19]. However, FDG-PET appears to be of limited usefulness in patients with osteosarcoma [20]. This might change with time as other positron emitter isotopes are developed.

Over the past 30 years, the 5-year survival for patients with osteosarcoma has dramatically improved from 10% to 70%. Surprisingly, however, the most effective regimen of chemotherapy uses the same agents that have been used for the past 20 years, namely, doxorubicin, cisplatin, methotrexate, and ifosfamide. Among treatment-related variables, only complete surgery has been reliably linked to improved survival. However, many contend that adjustments in the combination of chemotherapy and surgery are largely responsible [2].

## 4. Conclusion

Improvements in multimodal treatment for osteosarcoma, especially in the use of adjuvant chemotherapy, have increased event-free and overall survival. Meanwhile, the prolonged survival of patients has permitted the appearance of new significant, extra pulmonary targets for metastasis such as renal metastases. Ultimately, urologic, orthopedic, and general surgeons as well as radiologists and medical oncologists need to be thoroughly educated in regard to this entity because a multidisciplinary approach is ideal for these patients.
